# Effects of the epiretinal membrane on the outcomes of intravitreal dexamethasone implantation for macular edema secondary to branch retinal vein occlusion

**DOI:** 10.5935/0004-2749.20230011

**Published:** 2023

**Authors:** Ayse Gul Kocak Altintas, Cagri Ilhan

**Affiliations:** 1 Ankara Ulucanlar Eye Education and Research Hospital, University of Health Sciences, Ankara, Turkey.; 2 Hatay Education and Research Hospital, Hatay Turkey.

**Keywords:** Retinal vein occlusion/complications, Macular edema/ etiology, Tomography, optical coherence, Epiretinal membrane, Dexamethasone, Drug implants, Intravitreal injections, Oclusão da veia retiniana/complicações, Edema macular/etiologia, Tomografia de coerência óptica, Membrana epirretiniana, Dexametasona, Implantes de medicamento, Injeções intravítreas

## Abstract

**Purpose:**

To investigate the effects of epiretinal membrane formation on the clinical outcomes of intravitreal dexamethasone implantation for macular edema secondary to branch retinal vein occlusion.

**Methods:**

This retrospective interventional case series includes the treatment of naive patients with macular edema secondary to non-ischemic branch retinal vein occlusion who underwent intravitreal dexamethasone implantation. The patients were divided into two groups as follows: Group 1 (n=25), comprised of patients with macular edema secondary to branch retinal vein occlusion without epiretinal membrane, and Group 2 (n=16), comprised of patients with macular edema secondary to branch retinal vein occlusion with an epiretinal membrane. Corrected visual acuity, central macular thickness, and central macular volume values were measured before and after treatment. The clinical outcomes of the groups were compared.

**Results:**

Mean age and male-to-female ratio were similar between the two groups (p>0.05, for both). The baseline and final corrected visual acuity values, central macular thickness, and central macular volumes of the groups were similar (p>0.05, for all). All the parameters were significantly improved after intravitreal dexamethasone implantation treatment (p<0.001, for all). The changes in central macular thickness and volume were also similar (p>0.05, for both). The mean number of intravitreal dexamethasone implantations was 2.1 ± 1.0 (range, 1-4) in Group 1 and 3.0 ± 1.2 (range, 1-5) in Group 2 (p=0.043).

**Conclusion:**

Epiretinal membrane formation had no effects on the baseline and final clinical parameters, including corrected visual acuity and central macular thickness and volume. The only parameter affected by the presence of epiretinal membrane formation is the number of intravitreal dexamethasone implantations, a greater number of which is needed for macular edema secondary to branch retinal vein occlusion with an epiretinal membrane.

## Abstract (pt)

**Objetivo:**

Investigar os efeitos da formação de uma membrana epirretiniana nos resultados clínicos da implantação intravítrea de dexametasona para edema macular secundário à oclusão de um ramo da veia retiniana.

**Métodos:**

Esta série retrospectiva de casos intervencionais inclui o tratamento de indivíduos com edema macular secundário à oclusão não isquêmica de um ramo da veia retiniana, sem tratamento prévio e que foram submetidos a implantação intravítrea de dexametasona. Os indivíduos foram divididos em dois grupos: Grupo 1 (n=25), composto por indivíduos com edema macular secundário à oclusão de um ramo da veia retiniana sem a presença de uma membrana epirretiniana, e Grupo 2 (n=16), composto por indivíduos com edema macular secundário à oclusão de um ramo da veia retiniana com a presença de uma membrana epirretiniana. Os valores da acuidade visual corrigida, espessura macular central e volume macular central foram obtidos antes e após o tratamento. Os resultados clínicos dos grupos foram comparados.

**Resultados:**

A média de idade e a proporção entre homens e mulheres foram semelhantes nos dois grupos (p>0,05 para ambos os valores). Os valores iniciais e finais da acuidade visual corrigida, espessura macular central e volume macular central foram semelhantes nos dois grupos (p>0,05 para todos os valores). Todos os parâmetros melhoraram significativamente após o tratamento com implante de dexametasona intravítrea (p<0,001 para todos os parâmetros) e as alterações na espessura macular central e no volume macular central também foram semelhantes (p>0,05 para ambos os valores). O número médio de implantações intravítreas de dexametasona foi 2,1 ± 1,0 (faixa de 1-4) no Grupo 1 e 3,0 ± 1,2 (faixa de 1-5) no Grupo 2 (p=0,043).

**Conclusão:**

A formação de uma membrana epirretiniana não tem efeitos sobre os parâmetros clínicos iniciais e finais, incluindo a acuidade visual corrigida, a espessura macular central e o volume macular central. O único parâmetro afetado pela formação de uma membrana epirretiniana é o número de implantações intravítreas de dexametasona, sendo necessário um número maior de implantações em casos de edema macular secundário à oclusão de um ramo da veia retiniana com a presença de uma membrana epirretiniana.

## INTRODUCTION

Retinal vein occlusion (RVO) is one of the most common reasons for visual loss associated with retinal vascular disease^(1)^. It is prevalent in 1-2% of people aged >40 years, and the prevalence of branch RVO (BRVO) is four times greater than that of central RVO^(1)^. Macular edema (ME) is a common complication of BRVO and has the potential to permanently disrupt the macular architecture if left untreated^(2)^. In the past, treatment options were highly limited for ME secondary to BRVO^(3)^. Subsequent randomized controlled studies demonstrated improvement in the clinical outcomes of ME secondary to BRVO treated with intravitreal administrations of anti-vascular endothelial growth factors (anti-VEGFs) and steroids^(4,5,6,7,8)^. Intravitreal dexamethasone implantation (IDI) was found to be more effective than sham injections^(9)^. The Geneva study^(10)^ reported significant improvement in visual acuity in ME secondary to RVO.

An epiretinal membrane (ERM) is a disease of the vitreomacular interface involving both the macular and perimacular regions and can cause visual impairment or metamorphopsia. Anomalous posterior vitreous detachment resulting in vitreoschisis and vitreoretinal traction has been widely understood to be the most important pathophysiological mechanism^(11)^. Secondary ERM can be associated with inflammatory and retinal vascular diseases or retinal detachments^(12)^. The progression of ERM is generally slow and is not always clinically important; however, in association with other retinal conditions, mechanical vitreoretinal traction may change the course of underlying diseases and affect their treatment response^(12)^. The aim of this study was to investigate the effects of ERM formation on the anatomical and functional outcomes of IDI for ME secondary to BRVO by evaluating real-world data.

## METHODS

This retrospective interventional case series was conducted in a single tertiary referral hospital between June 2015 and June 2019. Approval was obtained from the local research ethics committee (Ankara Numune Education and Research Hospital). After a detailed explanation of the protocol, written informed consent was obtained before IDI was performed. All the procedures were performed in accordance with the ethical standards of the Declaration of Helsinki for human subjects.Medical records documenting the treatment of naive Caucasian patients with ME secondary to non-ischemic BRVO who underwent IDI as first-line therapy were investigated. The patient inclusion criteria were as follows: 1) age >18 years; 2) clinically (presence of intraretinal microvascular abnormalities or anastomotic vessels, localized retinal edema, venous dilation or sheathing within the retinal quadrant corresponding to the obstructed vein, and superficial or deep retinal hemorrhage)^(1)^ and angiographically (delayed arm-retinal transit time, late staining of the vein, non-perfusion or hyperpermeability of the retinal capillary bed in the ischemic area, petaloid pattern hyperfluorescence in the cystoid ME without macular ischemia associated with the increased foveal avascular zone)^(13)^ documented non-ischemic (non-perfusion area of the retinal capillary ≤5 disc-diameters on fluorescein angiography)^(14)^ BRVO history ≤6 months; 3) central macular thickness (CMT) >300 µm; 4) cataract surgery; and 5) at least 3 months of follow-up after IDI. The exclusion criteria were as follows: 1) history or clinical findings of other retinal diseases (e.g., diabetic retinopathy, age-related macular dystrophy, degenerative myopia, retinitis pigmentosa, or uveitis); 2) history of previous retinal treatment (e.g., vitrectomy, intravitreal injection or implantation, or laser photocoagulation); 3) history of increased intraocular pressure or anti-glaucomatous use and other risk factors of glaucoma (e.g., glaucoma history in a family member or thin central corneal thickness); 4) media opacity (e.g., corneal opacity, hyphema, or vitreous hemorrhage); 5) loss of vision due to other causes (e.g., neuroophthalmological diseases, retinal artery occlusion, or amblyopia); and 6) other reasons for secondary ERM (e.g., ocular trauma or primary vitreoretinal diseases).

Medical history and other ocular findings, including corrected visual acuity (CVA), were obtained from the patients’ medical records. CVA was determined using a Snellen chart, and the data were converted to logMAR. Colored fundus photographs, fundus autofluorescence, and fundus fluorescein angiograms were evaluated using a scanning laser ophthalmoscope (Heidelberg Retina Angiography 2, Heidelberg Engineering, Heidelberg, Germany). Macular configuration, vitreomacular interface, and quantitative analysis of CMT and central macular volume (CMV) were measured using spectral-do main OCT (Spectralis, Heidelberg, Germany), and quality scores ≥20 were considered acceptable. CMT was measured as the thickness of the central fovea, and CMV was measured in both the fovea and 6-mm perifoveal circular area. The patients were divided into two groups according to the presence or absence of ERM before treatment initiation. ERM was diagnosed as a hyperreflective membrane formation on the innermost layer of the retina on OCT. Group 1 was comprised of patients with ME secondary to BRVO without ERM, and Group 2 was comprised of patients with ME secondary to BRVO with ERM.

IDI (Ozurdex, Allergan, Inc., Irvine, CA, USA) was performed under sterile conditions as a first-line treatment for the all the patients. Then, the patients were instructed to use topical 0.5% moxifloxacin for a week. Retreatment was performed at least 3 months after the previous implantation if the ME persisted and the CVA did not improve as compared with the initial visit. Peripheric scatter retinal laser photocoagulation was applied in one or more sessions if evidence of peripheral retinal ischemia or neovascularization was found. Similarly, focal laser photocoagulation was performed if a focal ischemic area was observed close to the RVO region on angiography. IDI treatment was terminated in the following conditions: 1) complete ME regression and CVA stability in consecutive follow-ups; 2) incidence of adverse events, and 3) switching to another treatment option.

The Statistical Package for the Social Sciences (SPSS) 22.0 software (IBM Corp., New York, USA) was used for the statistical analysis. Descriptive data were presented as mean ± standard deviation (range). The Kolmogorov-Smirnov test was used to check the normal distribution of the variables. The Mann-Whitney *U* test was used to compare the groups, as the numerical data did not conform to a normal distribution. The statistical significance was set at p<0.05. The Sample Size Calculator software (ClinCalc LLC, Indianapolis, IN, USA) was used for the power analyses of the parameters, which showed significant differences.

## RESULTS

Group 1 included 25 eyes of 25 patients, and Group 2 included 16 eyes of 16 patients. The mean age of the patients was 54.6 ± 6.4 years (range, 44-68 years) in Group 1 and 59.3 ± 9.1 years (range, 46-71 years) in Group 2. The male-to-female ratio was 16:9 in Group 1 and 9:7 in Group 2. The demographic characteristics of the two groups were similar (p>0.05, for both).

Diabetes mellitus was the most common systemic comorbidity in both groups. Ten participants in Group 1 had diabetes mellitus, with a mean disease duration of 4.2 ± 3.6 years (range, 2-12 years), whereas five participants in Group 2 had diabetes mellitus, with a mean disease duarion of 5.5 ± 3.4 years (range, 2-11 years). Systemic hypertension and coronary artery diseases were the other most common systemic comorbidities after diabetes mellitus. The baseline clinical characteristics of the patients in the groups were similar (p>0.05, for all), as shown table 1.

**Table 1:** Baseline clinical characteristics of the groups

	Group 1	Group 2	p value
Diabetes mellitus (n)	10	5	0.575
Duration of diabetes mellitus (years)	4.2 ± 3.6 (2-12)	5.5 ± 3.4 (2-11)	0.412
Insulin use (n)	7	3	0.506
Oral antidiabetic use (n)	3	2	0.962
Systemic hypertension (n)	8	6	0.720
Antihypertensive use (n)	6	4	0.943
Coronary artery disease (n)	4	4	0.484
Antithrombotic use (n)	4	4	0.484
Other systemic comorbidities (n)	4	3	0.822
Endocrinological diseases (n)	2	1	0.836
Hematological diseases (n)	1	0	0.424
Rheumatological diseases (n)	1	0	0.424
Cerebrovascular diseases (n)	0	1	0.211
Malignity (n)	0	1	0.211

Peripheric scatter retinal laser photocoagulation was applied in one eye, and focal laser photocoagulation was also applied in one eye in Group 1. Focal laser photocoagulation was applied in one eye in Group 2. None of the eyes developed ERM after laser photocoagulation in Group 1 during the follow-up period.

**Figure 1:**
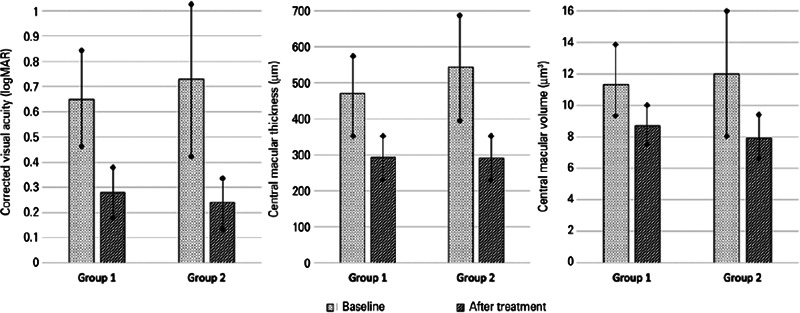
Changes of the patients’ clinical characteristics.

Before IDI treatment, the mean (range) CVA, CMT, and CMV were respectively 0.65 ± 0.2 logMAR (1.50-0.10 logMAR), 470.0 ± 126.7 µm (301-744 µm), and 11.3 ± 2.6 µm^3^ (8.7-21.1 µm^3^) in Group 1 and 0.73 ± 0.3 logMAR (1.50-0.10 logMAR), 543.1 ± 148.9 µm (384-855 µm), and 12.0 ± 4.0 µm^3^ (9.2-21.9 µm^3^) in Group 2. Despite the better baseline CVA, CMT, and CMV in Group 1, no significant differences were found between the two groups (p>0.05, for all). The mean (range) duration before the first IDI was 4.6 ± 1.0 months (3-6 months) in Group 1 and 4.1 ± 1.1 months (3-6 months) in Group 2, which were also similar (p>0.05). The clinical outcomes before IDI are given in table 2 and depictdd in figure 1.

**Table 2:** Summary of the clinical outcomes

	Group 1			Group 2			p value
	Before treatment	After treatment	p value	Before treatment	After treatment	p value	
CVA (logMAR)	0.65 ± 0.2 (1.50-0.10)	0.28 ± 0.1 (1.00-0.00)	<0.001	0.73±0.3 (1.50-0.10)	0.24 ± 0.1 (1.00-0.00)	<0.001	0.322* 0.642†
CMT (µm)	470.0 ± 126.7 (301-744)	294.1 ± 57.3 (216-416)	<0.001	543.1 ± 148.9 (384-855)	291.0 ± 61.0 (208-410)	<0.001	0.112* 0.885†
CMV (µm^3^)	11.3 ± 2.6 (8.7-21.1)	8.7 ± 1.4 (6.7-11.3)	<0.001	12.0±4.0 (9.2-21.9)	7.9 ± 1.5 (5.9-11.0)	<0.001	0.692* 0.900†
Change in CMT (µm)	176.0 ± 134.7 (6-463)			252.1 ± 151.6 (35-549)			0.112
Change in CMV (µm^3^)	2.6 ± 2.7 (1.0-10.6)			3.14 ± 3.13 (1.0-9.8)			0.149
Number of injections	2.1 ± 1.4 (1-4)			2.9 ± 1.3 (1-5)			0.043
Duration before the first implantation (months)	4.6 ± 1.0 (3-6)			4.1 ± 1.1 (3-6)			0.411
Duration between implantations (months)	4.1 ± 1.4 (3-6)			3.9 ± 1.3 (3-6)			0.289
Follow-up time after the first implantation (months)	8.7 ± 3.0 (3-22)			11.4 ± 3.9 (3-24)			0.245

CVA= corrected visual acuity; CMT= central macular thickness; CMV= central macular volume.

* Comparison of the pretreatment values between groups 1 and 2.

^†^Comparison of the posttreatment values between groups 1 and 2.

No complication was observed in any of the patients after or while receiving IDI. After IDI treatment, the mean (range) CVA, CMT, and CMV were respectively 0.28 ± 0.1 logMAR (1.00-0.00 logMAR), 294.1 ± 57.3 µm (216-416 µm), and 8.7 ± 1.4 µm^3^ (6.7-11.3 µm^3^) in Group 1 and 0.24 ± 0.1 logMAR (1.00-0.00 logMAR), 291.0 ± 61.0 µm (208-410 logMAR), and 7.9 ± 1.5 µm^3^ (5.9-11.0 µm^3^) in Group 2. The mean CVA, CMT, and CMV of the groups post IDI treatment were similar (p>0.05, for all). With IDI treatment, all the mean values of the parameters were significantly improved as compared with the pre-IDI treatment values (p<0.001, for all). The mean changes in CMT and CMV were similar between the two groups (p>0.05, for both). The clinical outcomes after IDI are presented in table 2 and illustrated in figure 1.

The mean follow-up time was 8.7 ± 3.0 months (range, 3-22 months) in Group 1 and 11.4 ± 3.9 months (range, 3-24 months) in Group 2 (p>0.05). The mean number of IDIs and duration between IDIs were 2.1 ± 1.0 months (range, 1-4 months) and 4.1 ± 1.4 months (range, 3-6 months) in Group 1 and 3.0 ± 1.2 months (range, 1-5 months) and 3.9 ± 1.3 months (range, 3-6 months) in Group 2, respectively. The mean number of IDIs was statistically higher in Group 2 (p=0.043), while the mean duration between IDIs was similar between the two groups (p>0.05). The clinical outcomes are summarized in table 2.

According to the power analysis results, in the comparison of two independent samples for the mean number of IDIs, a power of 80% could be attained if each group consisted of a minimum number of 16 patients (enrollment ratio 1:1, α=0.05, and β=0.2). The sample sizes of the groups in this study were in accordance with this condition.

## DISCUSSION

Currently, the most commonly used treatments for ME secondary to BRVO are intravitreal administration of anti-VEGF agents and dexamethasone. Many studies have compared the clinical outcomes of anti-VEGF and dexamethasone treatments. Comparable results have been reported in terms of anatomical and functional improvements, especially at mid- and long-term follow-ups^(15,16)^. Conversely, some adverse events such as cataract formation and intraocular pressure increase are more likely to occur after intravitreal dexamethasone treatment^(15,16)^. Therefore, many physicians conclude that dexamethasone treatment may be a more suitable alternative for pseudophakic patients, especially when considering the need for less frequent intravitreal administration^(15,16)^. In this study, IDI was preferred as a first-line treatment solely for pseudophakic patients without a history of intraocular pressure increases. Moreover, no persistent intraocular pressure increases requiring anti-glaucomatous medications were observed during the early- or long-term follow-up period in this study.

Pars plana vitrectomy, membrane peeling, and the intraocular gas tamponade injection protocol are generally considered standard treatment options for patients with symptomatic ERM, with generally quite satisfying anatomical outcomes^(17)^. Sometimes, residual intraretinal edema may persist, and adjuvant pharmacological therapy with intravitreal injection of steroid derivatives in addition to vitreoretinal surgery may accelerate the resolution of the associated intraretinal edema and hasten the recovery of visual function. This adjuvant therapy may be administered during or after surgery^(18,19,20)^. However, ERM-associated retinal comorbidities may better respond to less invasive treatments, and intravitreal pharmacotherapy may be used for some cases in place of vitreoretinal surgery. For instance, intravitreal anti-VEGF agent injection is accepted as a first-line therapy for diabetic ME patients with ERM formation^(21)^. Intravitreal drug administration may increase the vitreous volume due to vitreous liquefaction. Thus, spontaneous ERM separation may occurr, and this process is similar to the action mechanism of the mechanical relief of traction in pars plana vitrectomy^(22)^.

Baseline clinical characteristics are thought to be worse in ME patients with ERM. Mechanical vitreoretinal traction may increase CMT and CMV, and the optical barrier effect of a thicker membrane may play an additional role in decreasing CVA. Yiu et al.^(23)^ reported that patients with RVO-related ME had worse baseline CVA when ERM formation was present. In the study by Wong et al.^(24)^ ME caused by another etiology and similar baseline CVA and CMT values between diabetic ME with and without ERM were reported. In this study, the baseline clinical characteristics, including CVA, CMT, CMV, and duration before the first IDI, were similar between the two groups with and without ERM. Therefore, ERM was not an important additional risk factor for the worsening of baseline clinical characteristics or the need of earlier treatment in this study.

Only a limited number of studies have investigated the effects of intravitreal treatment on different patient groups with and without ERM. In diabetic ME patients with and without ERM, in the early term or mid-term after intravitreal anti-VEGF injection, the CVA and CMT results were not fully consistent. Maryam et al.^(25)^ reported that 1 month after intravitreal bevacizumab injection, CMT improved only in diabetic ME patients without ERM, and CVA improved only in diabetic ME patients with ERM. Ercalik et al.^(26)^ reported improvements in CVA and CMT in diabetic ME patients without ERM, but only CMT improved in diabetic ME patients with ERM at 3-month follow-up. Wong et al.^(24)^ reported worse CVA results 12 months after intravitreal ranibizumab therapy in diabetic ME patients with ERM. In neovascular age-related macular degeneration, which is another common indication for intravitreal treatment, limited responses to the intravitreal aflibercept injection may be observed in patients with and without ERM especially at short follow-up time points. Cho et al.^(27)^ reported worse 12-month CMT results in patients with neovascular age-related macular degeneration with ERM after they received intravitreal aflibercept injection. Follow-up time should be considered before evaluating the clinical response to intravitreal treatment in patients with and without ERM because early- and long-term outcomes may differ. In this study, the clinical parameters after IDI and their changes were similar between the patients with ME secondary to BRVO with and without ERM at a relatively long follow-up period. One important reason for the similar outcomes between the groups may be their similarities in baseline characteristics because baseline clinical parameters have been identified before as possible predictors of long-term outcomes^(28)^.

Similar to long-term outcomes, the need for a greater amount of intravitreal dexamethasone therapy may be predicted by considering some clinical data. Baseline visual acuity and early treatment response have been reported as two predictors in patients with ME secondary to BRVO^(28)^. In this study, the baseline clinical parameters and their changes were quite similar. However, the patients with ME secondary to BRVO with ERM formation needed more IDIs than the patients without ERM formation. Theoretically, the reason why some ME patients with ERM require more intravitreal treatments may be reduced perfusion of the drug into the retina due to the mechanical barrier effect of the ERM structure^(26)^. This hypothesis is compatible with the finding of this study because the only difference between the two groups was the presence of ERM formation.

Randomized controlled studies are considered a “gold standard” for clarifying the efficacy and safety of treatment modalities for any diseases. The outcomes of these studies are quite accurate because they include carefully selected, highly homogeneous study groups with strict treatment and follow-up schedules. However, the outcomes of real-world studies may not completely correlate with the outcomes of randomized controlled studies. In this regard, real-word data have better external validity despite having a lower certainty level^(29)^. The most important aspect of this study is that it directly reflects the long-term, real-world results of the effects of ERM on clinical outcomes after IDI for ME secondary to BRVO, which no other study has previously done as far as we know. Moreover, the evaluation of CMV is another essential aspect of this study. CMV is more associated with diffuse ME than with focal edema and can provide a more accurate information about the treatment outcomes in patients with ME^(30,31,32)^. The CMV results in this study could not be compared with those reported in the literature because, to the best of our knowledge, no previous studies have reported CMV results of patients with ME secondary to BRVO with ERM who underwent dexamethasone treatment. However, we observed that both the baseline and final CMVs and their changes were compatible with the values of the other clinical parameters. We presumed that CMV will be an important parameter for evaluating ME in future OCT-based studies.

This study has some limitations, including its small sample size (despite having a high statistical power) and retrospective design. Owing to being a real-world study and the longer efficacy period of dexamethasone than that of anti-VEGF agents, the posttreatment control time points during the follow-up period were not standardized for all the patients, and only baseline and final clinical data could be included in the statistical analyses. In addition, the ERM pattern and ellipsoid zone or the external limiting membrane integrity were not evaluated.

In conclusion, ERM formation had no effects on the baseline and final values of the clinical parameters, including CVA, CMT, and CMV, and on the changes in these parameters and the duration before the first IDI for ME secondary to BRVO. The only parameter affected by the presence of ERM formation was the number of IDIs, of which a more significant number is needed for ME secondary to BRVO with ERM.
